# Oxygen Desaturation Is Associated With Fibrocyte Activation *via* Epidermal Growth Factor Receptor/Hypoxia-Inducible Factor-1α Axis in Chronic Obstructive Pulmonary Disease

**DOI:** 10.3389/fimmu.2022.852713

**Published:** 2022-05-12

**Authors:** Chun-Hua Wang, Chun-Yu Lo, Hung-Yu Huang, Tsai-Yu Wang, Chih-Ming Weng, Chih-Jung Chen, Yu-Chen Huang, Fu-Tsai Chung, Chang-Wei Lin, Kian Fan Chung, Han-Pin Kuo

**Affiliations:** ^1^ Department of Thoracic Medicine, Chang Gung Memorial Hospital, Taipei, Taiwan; ^2^ College of Medicine, Chang Gung University, Taoyuan, Taiwan; ^3^ School of Respiratory Therapy, Taipei Medical University, Taipei, Taiwan; ^4^ Department of Pathology, Taichung Veterans General Hospital, Taichung, Taiwan; ^5^ School of Medicine, Chung Shan Medical University, Taichung, Taiwan; ^6^ Department of Thoracic Medicine, New Taipei Municipal TuCheng Hospital, New Taipei, Taiwan; ^7^ Airway Disease Section, National Heart and Lung Institute, Imperial College London, London, United Kingdom; ^8^ Biomedical Research Unit, Royal Brompton Hospital, London, United Kingdom; ^9^ Department of Thoracic Medicine, Taipei Medical University Hospital, Taipei, Taiwan; ^10^ School of Medicine, Taipei Medical University, Taipei, Taiwan

**Keywords:** chronic obstructive pulmonary disease (COPD), fibrocyte, epidermal growth factor receptor (EGFR), hypoxia-inducible factor-1α (HIF-1α), desaturation

## Abstract

Fibrocytes are bloodborne mesenchymal progenitors which accumulate and differentiate at the disease site. We investigated whether hypoxemia activates fibrocytes, accelerating airflow limitation and exercise intolerance in chronic obstructive pulmonary disease (COPD) patients. Flow cytometry was used to determine collagen I^+^/CD45^+^ fibrocytes and α-smooth muscle actin^+^ differentiating fibrocytes within peripheral blood and cultured cells, as well as the expression of CXC chemokine receptor 4 (CXCR4), epidermal growth factor receptor (EGFR), connective tissue growth factor (CTGF) and hypoxia-inducible factor (HIF)-1α. Fibrocytes in lung specimens were identified by confocal microscopy. Compared to non-desaturators, COPD desaturators (peripheral blood oxygen saturation ≤88% during exercise) had greater number of fibrocytes in peripheral blood and lung specimens, paralleled with faster yearly lung function decline and a 6-minute walk distance. Fibrocytes from desaturators expressed more EGFR, CXCR4, CTGF, and HIF-1α, with a higher capacity of proliferation and myofibroblastic differentiation. Hypoxia (5% oxygen) increased the expression of EGFR, CXCR4, CTGF, and HIF-1α, the number and differentiation in fibrocytes. These effects were attenuated by EGFR inhibitor gefitinib, HIF-1α gene silencing, and anti-CTGF antibody. These data elucidate that hypoxemia triggers fibrocyte activation through the EGFR/HIF-1α axis, aggravating airflow obstruction in COPD.

## Introduction

Fibrocytes are blood-borne progenitors with fibroblast properties, expressing mesenchymal protein type I collagen (COL I), leukocyte common antigen CD45, and hematopoietic stem cell antigen CD34 (1). Fibrocytes migrate towards disease sites under the guidance of chemokine gradients (2) and may subsequently transform into α-smooth muscle actin (α-SMA)-expressing myofibroblasts (3, 4). The role of circulating fibrocytes in the exacerbation and progression of airway obstruction has been recognized in the pathogenesis of asthma (2, 4). However, it is not known whether similar mechanisms operate in COPD.

Hypoxemia in COPD is caused by progressive airflow limitation and emphysematous destruction of the pulmonary capillary with subsequent ventilation to perfusion mismatching and could be further aggravated by obesity, sleep disorders, and exercise (5). Hypoxemia has been associated with systemic inflammation, skeletal muscle dysfunction, exercise intolerance, poor quality of life, and a shorter life expectancy in patients with COPD (6). Up to 39% of COPD patients who did not exhibit hypoxemia at rest experienced a fall in oxygen saturation (SpO_2_ ≤ 88%) during a 6-minute walk test (6MWT) (7). This group had frequent acute exacerbations, a significant decline in pulmonary function, and even an increased mortality (8). These observations suggest that intermittent desaturation during exercise could lead to tissue hypoxia that may result in a negative impact on COPD patients.

Hypoxia-inducible factor-1 (HIF)-1 is a heterodimeric protein consisting of an oxygen-regulated cytosolic α subunit and a constitutively-expressed nuclear β subunit (9). The HIF-1α subunit is rapidly degraded by ubiquitination *via* the proteasomal pathway, a process that is inhibited under hypoxic conditions (9). HIF-1α is the key regulator of the cellular response to hypoxia and is involved in hypoxia-induced chemokine receptor CXCR4 up-regulation and increased migratory activities in different cells, such as mononuclear phagocytes, endothelial cells, and cancer cells (10). Hypoxia also upregulates the protein and transcriptomic expression of epidermal growth factor receptor (EGFR), and activate tyrosine kinase (11, 12). EGFR up-regulation and activation contributes to increased proliferation and myofibroblast transformation in fibrocytes obtained from chronic obstructive asthma (13). EGFR activates HIF-1α and up-regulates the synthesis of HIF-1α (14–16). Moreover, HIF-1α is directly involved in hypoxia-induced connective tissue growth factor (CTGF) synthesis, a process that is independent of transforming growth factor-β1 (17).

In this study, we hypothesized that the number of circulating fibrocytes may be related to accelerated lung function decline in COPD patients with hypoxemia. Thus, we have investigated whether the fibrocytes of COPD patients, with exercise-induced hypoxemia, may be increased in the peripheral blood through up-regulation of CXCR4, and exhibit higher proliferation and myofibroblast transformation through up-regulation of EGFR, HIF-1α, and CTGF. This part of the study has been presented as an abstract to the 2019 ERS International Congress (18).

## Materials and Methods

### Patient Population

**Figure 1:**
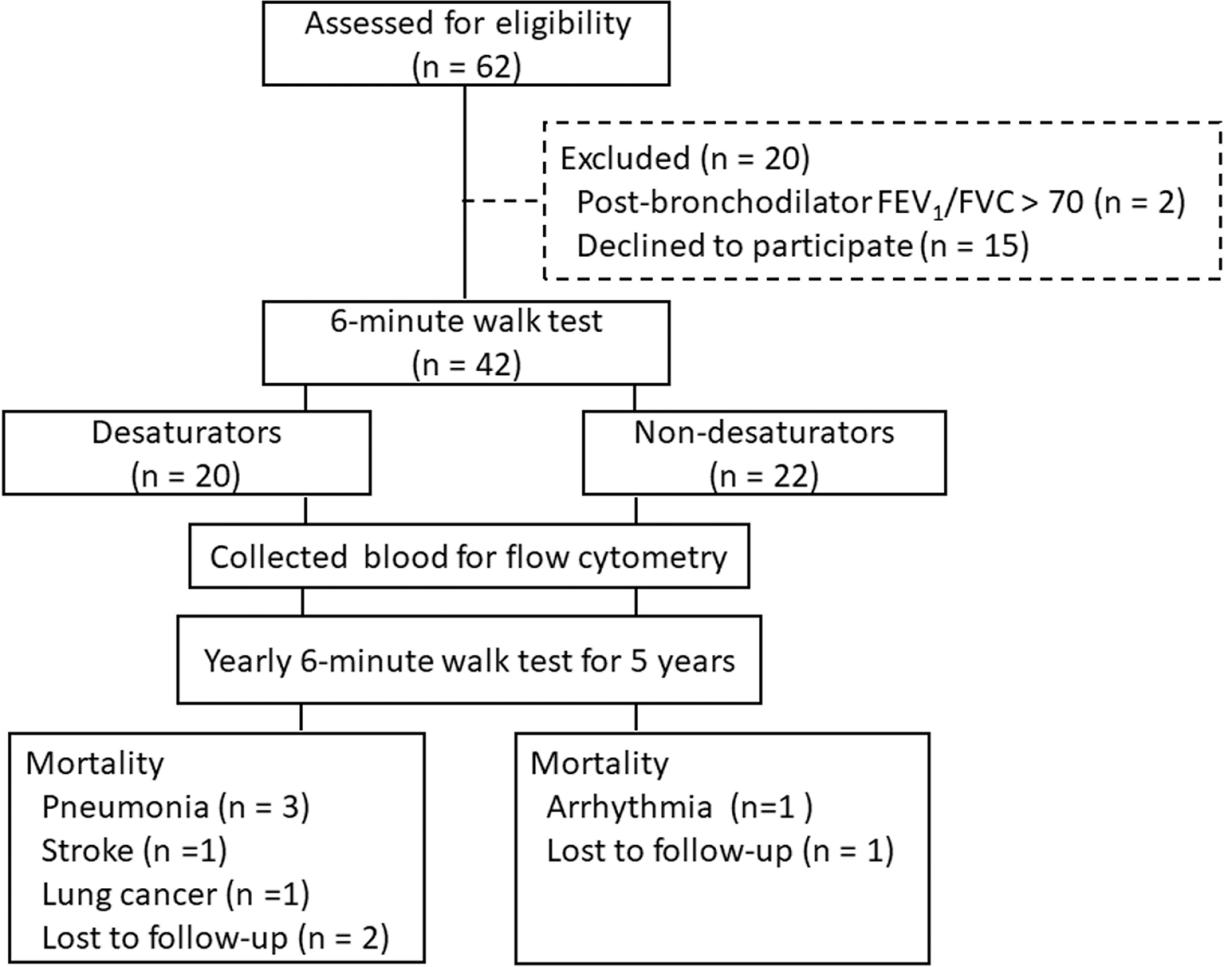
The flow chart of enrolled patients.

The study was performed using the COPD cohort of the Department of Thoracic Medicine, Chang Gung Memorial Hospital, Linkou, Taiwan, and the 5-year follow-up. Current or past smokers between 40 and 75 years of age were recruited. The diagnosis of COPD was confirmed by a post-bronchodilator forced expiratory volume in 1 second (FEV_1_)/forced vital capacity (FVC) ratio of less than 70% in the absence of a significant rise in FEV_1_ (12% and 200mL) after inhalation of fenoterol (19). Patients who had experienced an acute exacerbation of COPD or an upper airway infection in the preceding 2 months were not enrolled. The initial assessment for eligibility was 62 subjects and 20 were excluded (**Figure 1**). All 42 participants performed a 6-minute walk test (6MWT) to identify exercise-induced oxygen desaturation. A high-resolution computed tomography (HRCT) was performed to exclude alternative diagnoses such as bronchiolitis, bronchiectasis, cystic fibrosis, upper airways obstruction, or neoplastic diseases. Patients with a high anti-nuclear antibody titer (≥ 1:80), low complement protein C3 and C4, or evidence of systemic autoimmune diseases were excluded. Participants repeated 6MWT at least 2 months later to confirm the existence of exercise oxygen desaturation and then were further divided into non-desaturators (n = 22) and desaturators (n = 20). Desaturators were defined as patients with a persistent nadir SpO_2_ ≤ 88% during repeated 6MWTs. In contrast, non-desaturators were defined as patients with a persistent nadir SpO_2_ >89% during exercise. Tricuspid regurgitation gradient was accessed by Doppler echocardiography. Oxygen desaturation index (ODI, the number of ≥ 3% arterial oxygen desaturations per hour of sleep) was recorded by polysomnography (20). At the end of the 5-year follow-up, 7 of the desaturators and 2 of the non-desaturators passed away. The causes of mortality are listed in **Figure 1**. The study was approved by the Ethics Committee of Chang Gung Memorial Hospital (IRB: 98-3950B, 201801979A3). Written informed consents were obtained from all participants.

### Separation and Culture of Non-Adherent Non-T (NANT) Fraction of Peripheral Blood Mononuclear Cells (PBMCs)

Blood specimens were taken after 6MWTs. NANT cells were isolated as previously reported (2–4, 13, 21–23). Briefly, PBMCs were separated from whole blood using Ficoll-Hypaque density gradient centrifugation. The harvested PBMCs were washed and incubated in Iscove’s modified Dulbecco’s medium (IMDM; Sigma-Aldrich, Poole, UK) for 90 minutes to deplete the adherent monocytes and the T cells were depleted using CD3^+^ MicroBeads and magnetic separators according to the manufacturer’s instructions (Miltenyi Biotec, Auburn, CA).

The NANT cells were then be incubated in IMDM supplemented with 30% fetal bovine serum (Sigma-Aldrich, Poole, UK) and 1% bovine serum albumin either in a humidified incubator at 37°C with O_2_ 20%, CO_2_ 5% and N_2_ 75% (normoxic condition) or in an atmosphere-controlled culture chamber (Bellco Glass, Vineland, NJ) containing a gas mixture composed of O_2_ 5%, CO_2_ 5% and N_2_ 90% (hypoxic condition). Cells were treated with gefitinib (Tocris Bioscience, Bristol, UK; an inhibitor of EGFR) or anti-CTGF (Santa Cruz Biotechnology, Dallas, TX). Homemade small interfering RNA targeting HIF-1α (si-HIF-1α, 5′-AACUAACUGGACACAGUGUGU-3′, chosen corresponding to nt 661–681 of HIF-1α, GenBank^®^ accession number NM 001530) or scrambled siRNA control (si-Ctl) was transfected into NANT cells (25pmol per well) by using Lipofectamine RNAiMAX reagent (Invitrogen, Waltham, MA) following the manufacturer’s instructions. At 24 h, the cultured cells were harvested and assessed for the expression of HIF-1α in Collagen 1 (COL I+/CD45+ fibrocytes by means of flow cytometry (5). NANT cells were counted on a hemocytometer after trypan blue staining.

### Flow Cytometric Analysis

Cells were analyzed with BD FACScan flow cytometer equipped with an argon ion laser (Becton Dickinson Biosciences, San Jose, CA). Off-line analysis was performed using QUEST software as supplied by Becton Dickinson. Fibrocytes were defined as COL I^+^/CD45^+^ NANT cells by flow cytometry (2–4, 13, 20–22). Fibrocytes were verified by flow cytometry using both phycoerythrin (PE)-conjugated anti-CD34 (BD PharMingen, San Jose, CA), fluorescein isothiocyanate (FITC)-conjugated anti-collagen 1 antibodies (Chemicon International, Temecula, CA), and anti-CD45-peridinin chlorophyll protein complex (PerCp) polyclonal antibodies (BD PharMingen, San Jose, CA), as described previously (2–4, 13). NANT cells were fixed and immersed in 150 μl FACS permeabilizing solution (Becton Dickinson, San Jose, CA). The cell pellet was resuspended and incubated with FITC-conjugated anti-collagen 1 antibodies (Chemicon International, Temecula, CA), and then was incubated with anti-CD34-PE and anti-CD45-PerCP antibodies (BD PharMingen, San Jose, CA). To identify the expression of EGFR and CXCR4 on circulating fibrocytes, NANT cells were fixed first and incubated with PE-labelled anti-collagen 1 antibodies (Merck Milli-Mark, CA). Then, the cell pellet was incubated with anti-EGFR-FITC (Santa Cruz Biotechnology, Santa Cruz) and anti-CD45-PerCP polyclonal antibodies, or anti-CXCR4-FITC (Santa Cruz Biotechnology, Santa Cruz, CA) and anti-CD45-PerCp polyclonal antibodies. To determine the expression of HIF-1α in fibrocytes, NANT cells were stained with anti-COL I-FITC antibodies (Chemicon International, Temecula, CA), then followed by incubation with PE-labelled anti-human HIF-1α (R&D, Minneapolis, MN, USA) and anti-CD45-PerCp polyclonal antibodies. Permeabilised NANT cells were incubated with FITC-conjugated rabbit anti-human CTGF antibodies (Abcam, Cambridge, UK), followed by staining with anti-COL I-PE (Merck Milli-Mark, CA, USA) and anti-CD45-PerCp polyclonal antibodies to evaluate the expression of CTGF in the fibrocytes. Cells incubated with isotype-matched antibodies were used as negative controls.

The process of gating graph is described in **Supplementary Materials**.

### Immunofluorescence Staining for Confocal Microscopy

The paraffin-embedded lung tissue sections were obtained from 4 COPD desaturators and 4 COPD non-desaturators who underwent pleurodesis for pneumothorax. The tissue sections were placed onto glass slides, dewaxed in xylene and alcohols, and then washed twice with phosphate-buffered saline. The COL I was immunostained using a rabbit anti-human COL I primary antibody (Calbiochem, Darmstadt, Germany), followed by an Alexa-594–conjugated secondary antibody (Invitrogen, Carlsbad, CA). The CD34 was immunostained using anti-CD34-specific primary antibody (Millipore, Temecula, CA), then staining with Alexa-488-congugated secondary (Invitrogen, Waltham, MA). The Hoechst dye was used as a nuclear stain.

### Statistical Analysis

Statistical analysis was performed using Prism version 7.0 software package (GraphPad Prism Software Inc, San Diego, CA). Results were expressed as mean ± standard error of the mean (SEM). The differences between COPD non-desaturators and desaturators were estimated by a Mann-Whitney test. Intra-group comparisons, of two or more conditions, were carried out using the paired t-test and the Friedman test followed by Dunn’s *post-hoc* test, respectively. Correlations between parameters were determined by Spearman’s rank correlation. *P*-values of < 0.05 were accepted as statistically significant.

## Results

### Circulating Fibrocytes and Accelerated Decline in Lung Function and Exercise Tolerance in COPD Desaturators

**Figure 2:**
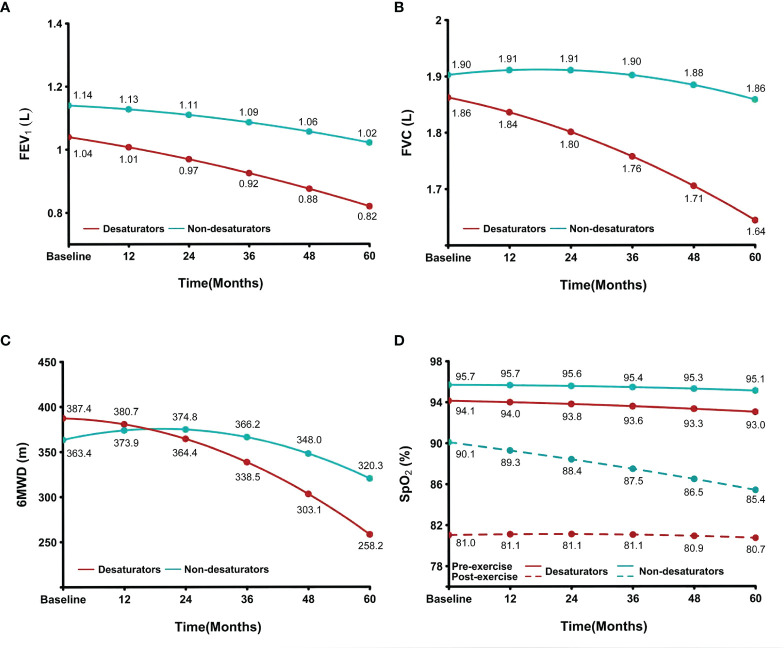
Accelerated reduction in oxygenation, lung function and exercise tolerance in COPD patients with exercise-induced hypoxemia. The data of **(A)** FEV_1_, **(B)** FVC, **(C)** 6MWD and **(D)** SpO_2_ at the point of baseline, 12, 24, 36, 48 and 60 months between non-desaturators (n = 84) and desaturators (n = 86) were indicated in the image of COPD cohort over a 5-year period. SpO_2_, peripheral blood oxygen saturation; FEV1, forced expiratory volume in 1 second; FVC, forced vital capacity; 6MWD, 6-minute walking distance; COPD, chronic obstructive pulmonary disease; L, liter; m, meter.

The change in clinical presentation of 170 subjects in our COPD cohort are shown in **Figure 2**. Compared to COPD non-desaturators (n = 84), those desaturators (n = 86) had a greater reduction in lung function and exercise tolerance during 6MWT at Year 5. The post-exercise oxygenation of non-desaturators declined progressively during the 5-year follow-up period but was still higher that of non-desaturators.

Circulating fibrocytes were isolated from blood samples of COPD non-desaturators (n = 22) and desaturators (n = 20) and the clinical data are summarized in **Table 1**. Because the specimens collected from single patients were not sufficient for all experiments, we only used blood from some participants for some experiments as proof of concept. There was no significant difference in lung function, walking distance at baseline (Year 0), and doppler-determined peak tricuspid regurgitation gradient between non-desaturators and desaturators. Both groups had a normal SpO_2_ at rest, but COPD desaturators as defined, showed severe hypoxemia after exercise (91.0 ± 0.6% versus 77.0 ± 2.0%, *P* < 0.0001). Compared to non-desaturators (ODI 7.7 ± 2.7/hr), desaturators had more frequent oxygen saturation during sleep (ODI 30.2 ± 7.4/hr, *P* = 0.0083). The ODI value was positively correlated with the extent of post-exercise reduction in SpO_2_ (r_s_ = 0.539, *P* = 0.0086). During the 5-year follow-up, both groups had a decreased FEV_1_/FVC ratio but desaturators had greater reduction in 6-min walk distance and FEV_1_.

**Table 1:** Clinical characteristics of study subjects.

	Non-Desaturators		Desaturators	
Male/Female	22/0		20/0	
Age, years	71.1 ± 1.3		68.3 ± 1.8	
BMI, kg/m2	24.0 ± 0.7		21.9 ± 1.1*	
Smoking, pack-year	40.0 ± 6.0		51.3 ± 7.3*	
ODI, %	7.7 ± 2.7		30.2 ± 7.4**	
Lung function	Year 0	Year 5	Year 0	Year 5
FVC, L	1.99 ± 0.17	2.17 ± 0.18	1.87 ± 0.13	1.72 ± 0.13
FVC %Pred	63.2 ± 4.5	70.7 ± 4.8†	60.4 ± 4.8	59.2 ± 4.5
FEV_1_, L	1.14 ± 0.10	1.13 ± 0.11	0.96 ± 0.08	0.80 ± 0.07*††
FEV_1%_Pred	51.1 ± 4.5	49.3 ± 4.8	42.4 ± 4.1	37.5 ± 3.2*†
FEV_1_/FVC, %	58.0 ± 2.6	51.0 ± 2.5††	54.2 ± 2.1	45.7 ± 2.4††
6-minute walk test				
Pre-Ex SpO_2_, %	95.3 ± 0.3	94.4 ± 0.4	95.1 ± 0.4	94.1 ± 0.9
Post-Ex SpO_2,_ %	91.0 ± 0.6	87.2 ± 1.5†	77.0 ± 2.0***	82.5 ± 2.0
6MWD, m	345.6 ± 20.6	383.1 ± 19.6	377 ± 26.3	285.3 ± 39.0†
TRPG, mmHg	37.5 ± 1.2	37.0 ± 1.3	31.1 ± 2.7	33.4 ± 3.2

Paired or unpaired t-test *P < 0.05, **P < 0.01 and ***P < 0.0001 versus non-desaturators, †P < 0.05, ††P < 0.01 versus Year 0. BMI, body mass index; ODI, oxygen desaturation index; L, liter; FEV_1_, forced expiratory volume in 1 second; FVC, forced vital capacity; %pred, percent of predicted value; Ex, exercise; SpO_2_, peripheral blood oxygen saturation; 6MWD, 6-minute walk distance; m, meter; TRPG, tricuspid regurgitation peak gradient.

**Figure 3:**
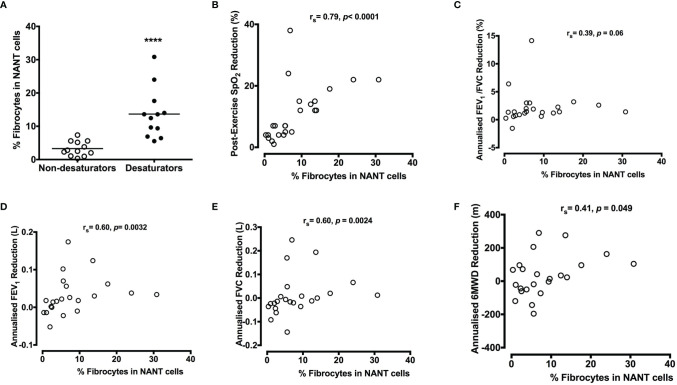
Higher percentage of circulating fibrocytes with greater lung function decline in COPD desaturators **(A)**. NANT cells were isolated from peripheral blood of non-desaturators (n = 12) and desaturators (n = 12) and the percentage of collagen I^+^/CD45^+^ fibrocyte in freshly isolated NANT cells on day 0 was determined by flow cytometry. Horizontal lines represent the mean values for each group. **(B–F)** All participants performed 6-minute walking tests at the beginning and during the 5-year follow-up. The correlation between the percentage of fibrocytes and the extent of post-exercise reduction in SpO_2_ at baseline **(B)**, annualized change in FEV_1_/FVC ratio **(C)**, FEV_1_ **(D)**, FVC **(E)** and 6MWD **(F)** are shown. The differences between non-desaturators and desaturators were determined by Mann-Whitney test and the correlation was determined by Spearman’s rank correlation. ****P < 0.0001. COPD, chronic obstructive pulmonary disease; NANT cells, non-adherent non-T fraction of peripheral blood mononuclear cells; FEV_1_, forced expiratory volume in 1 second; FVC, forced vital capacity; %pred, percent of predicted value; Ex, exercise; SpO_2_, peripheral blood oxygen saturation; 6MWD, 6-minute walking distance.

The proportion of circulating fibrocytes in NANT cells (day 0 at Year 0) was significantly increased in COPD desaturators (13.7±2.2%, n=12) compared to that in COPD non-desaturators (3.3±0.6%, n=12, *P <*0.0001) (**Figure 3A**). The percentage of fibrocytes in NANT cells was positively correlated with the extent of post-exercise reduction in SpO_2_, and yearly decline rate in FEV_1_, FVC, and 6MWT distance (**Figures 3B–F**). Higher ODI was also associated with more fibrocytes in these COPD participants (r_s_ = 0.55, *P* = 0.0059).

### Fibrocytes in Lung Tissue of COPD Desaturators

**Figure 4:**
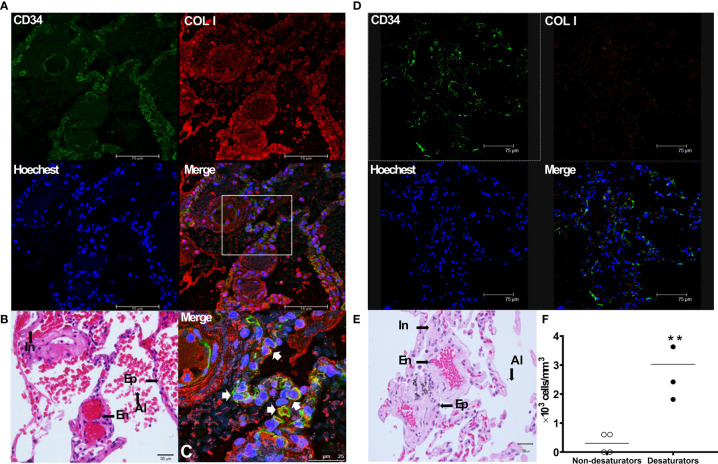
Identification of COL I^+^/CD34^+^ fibrocytes in the lung tissue of COPD desaturators **(A–C)**. The paraffin sections of lung tissue from COPD desaturators were stained for the CD34 surface marker (*green*), intracellular COL I (*red*), and nuclei (*blue*) **(A)**, and for hematoxylin and eosin staining **(B)**. The immunofluorescence staining and confocal microscopic analysis are described in Methods. **(C)** Shows a magnification of the merged image of **(A)**, and *arrows* indicate the double staining of COL I ^+^/CD34^+^fibrocytes with yellow enhancement in the merged image. *Bar*, 75 μm **(A)** and 25μm **(C)**. **(D, E)** The paraffin sections of airway tissue from COPD non-desaturators were stained for indicated immunofluorescence staining **(D)** and hematoxylin and eosin staining **(E)**, as described above. *Bar*, 75 μm **(D)** and 30 μm **(E)**. **(F)** The differences between the numbers of fibrocytes in specimens from non-desaturators and desaturators were determined by Mann-Whitney test. **P < 0.01. COL I, collagen I; COPD, chronic obstructive pulmonary disease; In, interstitium; Ep, epithelium; En, endothelium; Al, alveoli.

Immunofluorescence staining was performed on paraffin-embedded lung biopsy specimens to identify the presence of fibrocytes in lung tissue from COPD desaturators and non-desaturators. As shown in **Figure 4**, fibrocytes with a dual expression of COL I^+^/CD34^+^(merged in yellow in **Figure 4C**) were identified in the bronchioles, bronchio-alveolar ducts, interstitium, and alveolar epithelial areas in COPD desaturators. There were much fewer fibrocytes in the COPD non-desaturators (**Figures 4D, E**).

### Increased Expression of CXCR4 on Fibrocytes of COPD Desaturators

**Figure 5:**
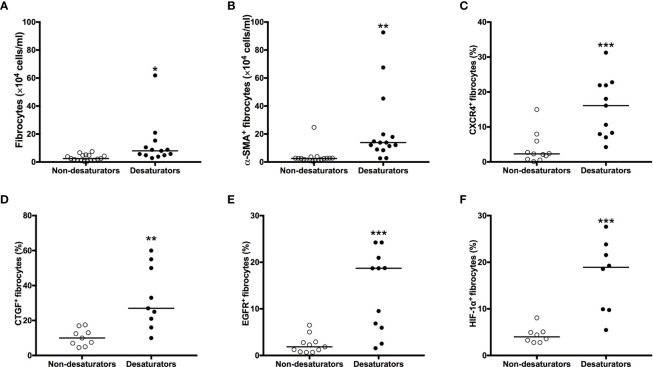
Higher CXCR4, CTGF, EGFR and HIF-1α expression with greater increased in number and myofibroblastic differentiation in fibrocytes from COPD desaturators. The number of Col I^+^/CD45^+^ fibrocytes **(A)** and differentiating α-SMA^+^ fibrocytes **(B)** in NANT cells were determined by flow cytometry after 14 days in culture. NANT cells were isolated from COPD non-desaturators and desaturators. The proportion of CXCR4- **(C)**, CTGF- **(D)**, EGFR- **(E)** and HIF-1α- **(F)** expressing fibrocytes within freshly isolated NANT cells from desaturators, determined by flow cytometry, was higher compared to that from non-desaturators. Horizontal lines represent the median values for each group. The differences between disease groups were determined by Mann-Whitney test. *P < 0.05, **P < 0.01, ***P < 0.001. CXCR4, CXC chemokine receptor 4; CTGF, connective tissue growth factor; EGFR, epidermal growth factor receptor; HIF-1α, hypoxia-inducible factor-1α; COPD, chronic obstructive pulmonary disease; NANT cells, non-adherent non-T fraction of peripheral blood mononuclear cells; α-SMA, α-smooth muscle actin.

To determine the mechanisms for increased mobilization of fibrocytes in COPD desaturators, the surface expression of CXCR4 in peripheral blood fibrocytes were identified by flow cytometry. There was an increased proportion of circulating fibrocytes expressing CXCR4 in desaturators compared to non-desaturators (**Figure 5C**). Fibrocytes from non-desaturators exposed to hypoxic conditions (5% O_2_) for 2 h had a greater proportion of CXCR4-expressing fibrocytes compared with those incubated in normoxic condition (20% O_2_) (**Figure 5D**).

### Increased Number and Myofibroblastic Differentiation and Higher Expression of EGFR, CTGF, and HIF-1α of Fibrocytes From COPD Desaturators

To compare the number and differentiative capacities of fibrocytes between COPD non-desaturators and desaturators, 1×10^6^ NANT cells per well were seeded at day 0. There was a significant increase in the number of fibrocytes as well as myofibroblast differentiating (α-SMA^+^) fibrocytes from COPD desaturators but not in that from non-desaturators after 14 days of culture, respectively (**Figures 5A, B**).

To explore the underlying mechanisms, peripheral blood NANT cells were analyzed by flow cytometry for their expression of CTGF and EGFR. There was an increased number of circulating fibrocytes expressing both CTGF and EGFR in COPD desaturators when compared with non-desaturators (**Figures 5D, E**). A higher proportion of circulating fibrocytes from COPD desaturators was positive for HIF-1α expression (25.4 ± 6.1%) compared to that of non-desaturators’ fibrocytes (11.8 ± 5.0%, *P* < 0.001) (**Figure 5F**).

### Hypoxia Increased Number, Differentiation, and CXCR4 Expression Through EGFR/HIF-1α Axis in Fibrocytes

**Figure 6:**
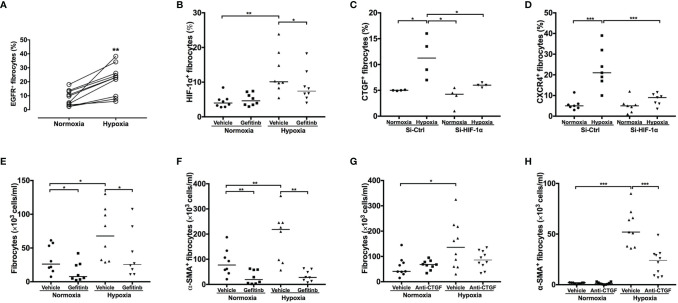
EGFR and HIF-1α inhibition and anti-CTGF antibodies blocked hypoxia-induced fibrocyte activation. NANT cells from COPD non-desaturators were exposed to normoxic (O_2_ 20%) or hypoxic (O_2_ 5%) condition for 24h and incubated in the presence of gefitinib (1 μμ) and anti-CTGF antibodies (1 μg/ml). The expression of EGFR at 24h **(A)**, HIF-1α **(B)**, CXCR4 **(C)** and CTGF **(D)** at 2h and the number of fibrocytes **(E, G)** and α-SMA^+^ differentiating myofibroblasts **(F, H)** at 14 days were determined by flow cytometry. Horizontal lines represent the median values for each group *P < 0.05, **P < 0.01, ***P < 0.001. EGFR, epidermal growth factor receptor; HIF-1α, hypoxia-inducible factor-1α; CTGF, connective tissue growth factor; NANT cells, non-adherent non-T fraction of peripheral blood mononuclear cells; COPD, chronic obstructive pulmonary disease; CXCR4, CXC chemokine receptor 4; si-Ctl, siRNA control; α-SMA, α-smooth muscle actin.

**Figure 7:**
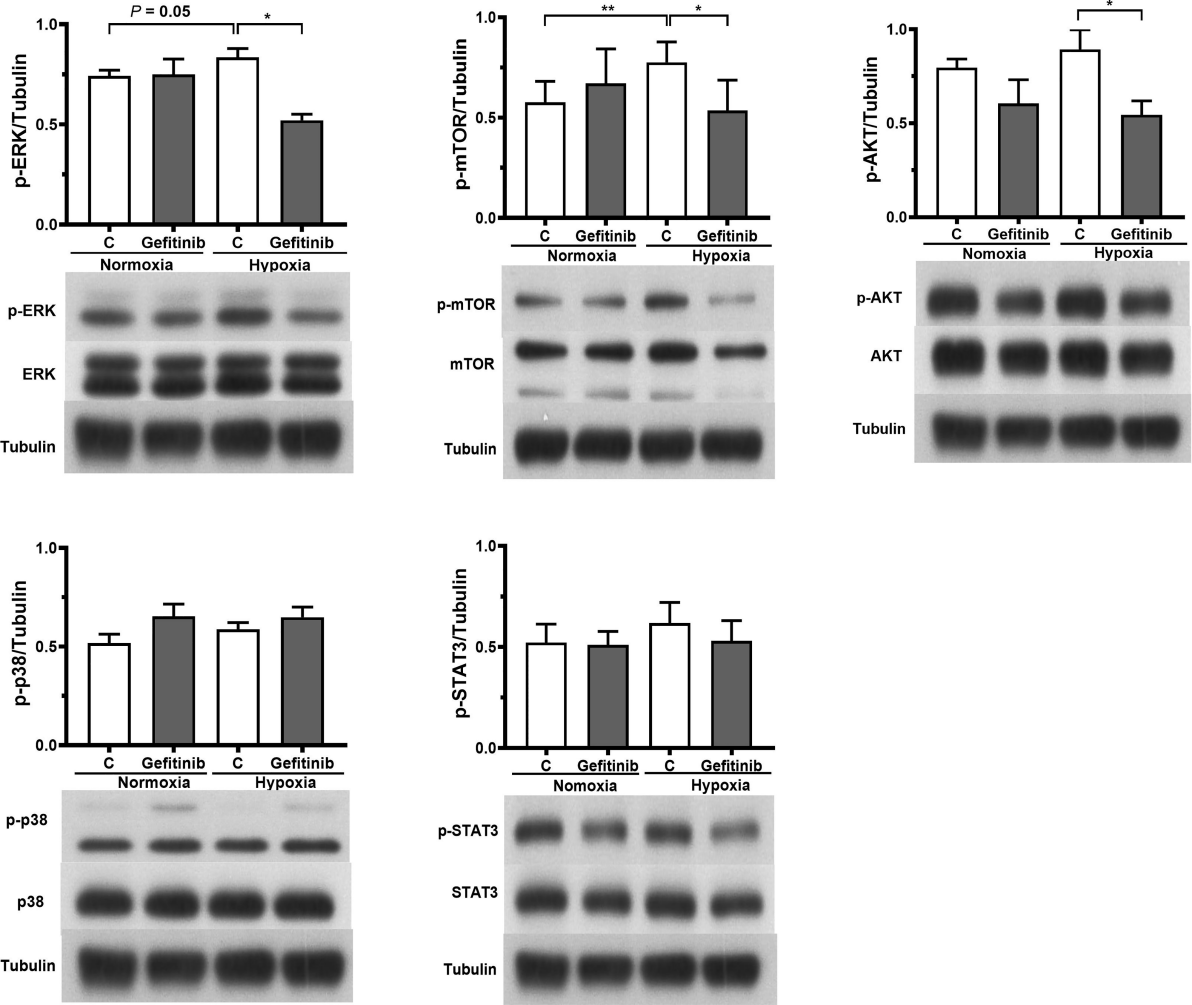
Western blot analysis was used to quantify the expression of MAPK kinase (p-38), extracellular signal-regulated kinase (ERK), phosphatidylinositol-3-kinase/protein kinase B (PI3K/AKT), signal transducer and activator of transcription-3 (STAT-3), and mammalian target of rapamycin (mTOR) in MRC-5 human fetal lung fibroblasts (n = 4), which were exposed to normoxic (O_2_ 20%) or hypoxic (O_2_ 5%) condition and in the absence or presence of gefitinib (1 μM) for 24 hours. *P < 0.05, **P < 0.01 compared with control. C, control; p-, phospho-.

Exposure to hypoxic conditions significantly increased the number of non-desaturator’s fibrocytes expressing EGFR and HIF-1α (**Figures 6A, B**). Treatment with the EGFR tyrosine kinase inhibitor, gefitinib (1 μM), inhibited hypoxia-induced HIF-1α expression (**Figure 5B**). Hypoxia increased the expression of CTGF and CXCR4 in non-desaturators’ fibrocytes, and the effect of hypoxia could be suppressed by si-HIF-1α knockdown (**Figures 6C, D**). Under the hypoxic condition, the EGFR down-stream signal transduction pathways, both extracellular signal-regulated kinase (ERK) and the serine/threonine kinase AKT/mammalian target of rapamycin (mTOR), were activated which were blocked by treatment with the EGFR tyrosine kinase inhibitor, gefitinib (**Figure 7**). However, the p38 and activator of transcription-3 (STAT-3) pathways were not activated or affected by the treatment of gefitinib (**Figure 7**).

Treatment with Gefitinib (1 μM) reduced the number and suppressed myofibroblastic transformation of fibrocytes induced by hypoxia in fibrocytes of COPD non-desaturators (**Figures 6E, F**). Treatment with an anti-CTGF antibody (1 μM) notably inhibited hypoxia-increased myofibroblast transformation but not proliferation (**Figures 6G, H**).

## Discussion

**Figure 8:**
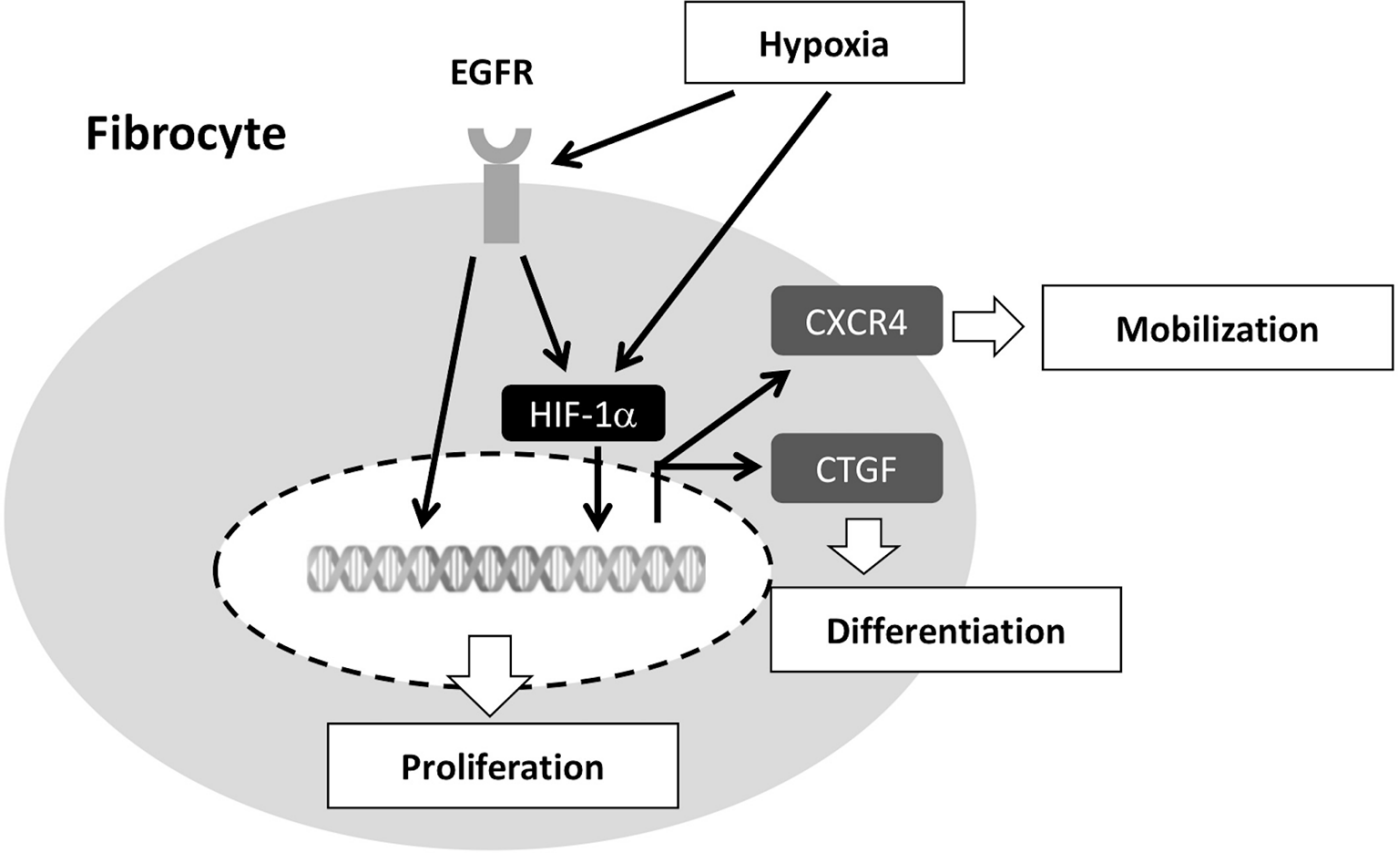
Proposed molecular mechanisms involved in hypoxia-induced fibrocyte mobilization, proliferation, and differentiation. Hypoxia up-regulates EGFR, HIF-1α, and CTGF. Hypoxia-induced EGFR transactivation promotes the transcription of HIF-1α-targeted genes (e.g. CTGF and CXCR4). Elevated HIF-1α increases CXCR4 and CTGF expression, leading to increased number of fibrocytes recruited into the circulation and differentiated into myofibroblasts. EGFR, epidermal growth factor receptor; CXCR4, CXC chemokine receptor type 4; CTGF, connective tissue growth factor; HIF-1α, hypoxia-inducible factor-1α.

We have previously shown the role of circulating fibrocytes in the pathobiological mechanisms of asthma. First, increased fibrocyte numbers with higher expression of chemokine receptors (i.e., CXCR4 and CCR7) have been observed in asthmatic patients during acute exacerbation and in those with more significant airflow limitation (2). Circulating fibrocytes in patients with chronic obstructive asthma, as compared to those from mild asthma, possess a higher capacity for proliferation and myofibroblastic transformation and these patients have a more rapid decline in pulmonary function (4). Up-regulated expression of EGFR and of CTGF have been linked to the higher proliferative and transformative capacities of fibrocytes (3, 13). In present study, we further demonstrated that intermittent hypoxemia in COPD patients was accompanied with exercise intolerance and faster lung function decline that was related to recruitment and activation of circulating fibrocytes. In the lung tissues from our COPD patients with concomitant pneumothorax, fibrocytes were mostly identified in small airways and the interstitium of desaturators but not in non-desaturators. Compared to COPD non-desaturators, COPD desaturators in our study had a greater number of fibrocytes in the peripheral blood that exhibited a higher expression of EGFR, HIF-1α and CTGF, resulting in increased proliferation and myofibroblastic differentiation. The proposed mechanisms associated with hypoxia-induced mobilization, proliferation, and differentiation is illustrated in **Figure 8**.

The rate of FEV_1_ loss is one of the most important parameters for COPD progression. Emphysema severity measured by CT and current smoking status have been validated as clinical parameters that would identify the rapid lung function decliners in COPD (24). Our cohort and previous data have reported a significant reduction of FEV_1_ in COPD patients with exertional desaturation over a 3- to 8-year follow-up period (25, 26). The number of circulating fibrocytes have been shown to be positively correlated with the severity of airflow limitation in chronic obstructive asthma and in severe asthma (2, 4, 21). The number of circulating fibrocytes is also increased during an acute exacerbation of COPD and is associated with higher mortality and negatively correlated with FEV_1_, FVC, FEV_1_/FVC ratio, diffusion capacity, and oxygen saturation (22). An increased number of fibrocytes has been found in the bronchial specimens from COPD patients and the density of fibrocytes is negatively associated with lung function and positively correlated with bronchial wall thickness quantified by computed tomographic scans (22). The present study not only supported a faster lung function decline in COPD desaturators, but also demonstrated a close relationship between the number of fibrocytes in peripheral blood and the severity of exercise-induced hypoxemia, decline rate of FEV_1,_ and the 6MWT distance.

Up-regulation of CXCR4 in fibrocytes of patients with chronic obstructive asthma is essential for mobilization of bone marrow-derived fibrocytes into the circulation (2). CXCR4 is the surface receptor for CXCL12 (stromal cell derived factor-1α). CXCR4/CXCXL12 axis has been shown to induce the directional migration of progenitor cells in the bone marrow to the injured tissues and is involved in many biological responses, including hematopoiesis, immune response, and cancer metastasis (27). Fibrocytes collected during exacerbation of COPD and asthma had increased chemotactic migration in response to CXCL12 (22). Both CXCR4 and CXCL12 are up-regulated by hypoxia and by activation of HIF-1α (10). In this study, the circulating fibrocytes in COPD desaturators were found to highly express CXCR4 and HIF-1α. Our study also showed that under hypoxic conditions, fibrocytes of COPD non-desaturators had increased CXCR4 expression. Knocking down HIF-1α by RNA interference led to the suppression of hypoxia-induced CXCR4 expression. In the lung tissues of our COPD patients who underwent pleurodesis for pneumothorax, fibrocytes were mostly identified in the small airways and pulmonary interstitium of COPD desaturators. Thus, under hypoxic condition, fibrocytes from COPD may mobilize into the circulation and migrate to small airways by increasing HIF-1α-induced CXCR4 expression, and then contribute to airflow limitation.

HIF-1α protein synthesis is regulated by activation of the phosphatidylinositol 3-kinase (PI3K) and extracellular signal-regulated kinase pathways. These pathways can be activated by signaling *via* receptor tyrosine kinases, non-receptor tyrosine kinases, or G-protein-coupled receptors (28). We showed that inhibition of EGFR by gefitinib reduced hypoxia-induced HIF-1α expression, compatible with the previous report showing a decrease in hypoxia-induced accumulation of HIF-1α after EGFR inhibition in human lung cancer A549 cells (29). In COPD desaturators, EGFR expression was up-regulated in circulating fibrocytes. Under hypoxic condition, EGFR expression increased in fibrocytes of COPD non-desaturators, implying that up-regulated EGFR expression in COPD desaturators can be attributed to hypoxia. Some hypoxia-inducible transcriptional regulators, such as early growth response-1 (EGR-1), enhance the basal transcriptional activity of the EGFR promoter (30, 31). A non-coding genetic variant rs6593210 which disrupts the hypoxia-response element (HRE) also transcriptionally regulates EGFR in patients with COPD and obstructive sleep apnea (32). We have previously demonstrated that EGFR up-regulation and activation increased fibrocyte proliferation and differentiation in chronic obstructive asthma patients (13). Fibrocytes from COPD desaturators had a greater capacity to proliferate and differentiate into myofibroblasts which can be attributed to the up-regulated expression of EGFR in fibrocytes. In this study we also showed that the EGFR inhibitor, gefitinib, suppressed hypoxia-induced fibrocyte proliferation and myofibroblast transformation. The EGFR pathway is complex. Our findings revealed that the activation of EGFR in the fibrocytes exposed to hypoxic status activated the EGFR down-stream signal pathways of both ERK and AKT/mTOR but not STAT-3 or p38. Both ERK and AKT/mTOR are important regulators for cell proliferation and survival (33). Additionally, the ERK pathway is implicated in hypoxia-induced CTGF synthesis (34).

CTGF is over-expressed in fibrocytes from chronic obstructive asthmatic patients and CTGF plays an important role in fibrocyte differentiation (3). Up-regulation of CTGF was found in circulating fibrocytes of COPD desaturators and hypoxia increased CTGF expression in fibrocytes from COPD non-desaturators. Treatment with anti-CTGF antibodies did not significantly inhibited hypoxia-induced fibrocyte proliferation but inhibited myofibroblast transformation, suggesting that CTGF expression was more related to fibrocyte differentiation than proliferation. HIF-1α is directly involved in CTGF expression at the transcriptional level (17). In our study, transfection with siRNA against HIF-1α significantly suppressed hypoxia-induced CTGF expression. Thus, in COPD desaturators, the increased expression of CTGF in circulating fibrocytes is mediated *via* the up-regulated HIF-1α.

In conclusion, COPD patients with exercise-induced oxygen desaturation have an increased number of circulating fibrocytes with a heightened myofibroblastic transformation, possibly mediated by the EGFR-HIF-1α axis. Elevated HIF-1α expression increased CXCR4 and CTGF expression, contributing to an increased number of fibrocytes recruited into the circulation that possess an increased differentiating capacity into myofibroblasts. Inhibition of EGFR and HIF-1α signaling could represent a rational strategy for novel therapeutic development of fibrotic responses. Our findings uncover the role of fibrocytes in COPD with exercise-induced hypoxemia and our research model may be extrapolated to hypoxia-induced end-stage fibrosis of multiple organs including lungs, heart, kidneys, and liver.

## Data Availability Statement

The original contributions presented in the study are included in the article/**Supplementary Material**. Further inquiries can be directed to the corresponding authors.

## Ethics Statement

The studies involving human participants were reviewed and approved by Ethics Committee of Chang Gung Memorial Hospital (IRB: 98-3950B, 201801979A3). The patients/participants provided their written informed consent to participate in this study.

## Author Contributions

C-HW and C-YL performed the experiments, C-HW and H-PK designed the experiments, C-YL and KC interpreted the data, C-HW and C-YL wrote the first draft, C-JC reviewed the lung tissue specimens, H-YH, T-YW, C-MW, Y-CH, F-TC, and C-WL participated in the interpretation of the data, C-HW and H-PK devised the study, KC discussed the findings, C-HW and H-PK finalized the manuscript. All authors contributed to the article and approved the submitted version.

## Funding

This work was supported by the National Medical Research Program (NMRP) grant 99-2314-B-182-043-MY3, 108-2314-B-182A-128-MY2 and the Chang Gung Medical Research Project (CMRP) grant CMRPG390041-2.

## Conflict of Interest

The authors declare that the research was conducted in the absence of any commercial or financial relationships that could be construed as a potential conflict of interest.

## Publisher’s Note

All claims expressed in this article are solely those of the authors and do not necessarily represent those of their affiliated organizations, or those of the publisher, the editors and the reviewers. Any product that may be evaluated in this article, or claim that may be made by its manufacturer, is not guaranteed or endorsed by the publisher.
