# Supplementary material for: Oxygen Desaturation Is Associated With Fibrocyte Activation via Epidermal Growth Factor Receptor/Hypoxia-Inducible Factor-1α Axis in Chronic Obstructive Pulmonary Disease
Source: Front Immunol. 2022 May 12;13:852713. doi: 10.3389/fimmu.2022.852713 (PMC9134242; doi:10.3389/fimmu.2022.852713)
Supplement: Supplementary file 1 [file DataSheet_1.docx]

**Supplementary Material**

**Oxygen desaturation is associated with fibrocyte activation via epidermal growth factor receptor/hypoxia-inducible factor-1α axis in chronic obstructive pulmonary disease**

Chun-Hua Wang^1^*^†^, Chun-Yu Lo^1†^, Hung-Yu Huang^1^, Tsai-Yu Wang^1^, Chih-Ming Weng^2^, Chih-Jung Chen^3^, Yu-Chen Huang^1^, Fu-Tsai Chung^1,4^, Chang-Wei Lin^1^, Kian Fan Chung^5^, Han-Pin Kuo^6^*

^1^ Department of Thoracic Medicine, Chang Gung Memorial Hospital, Taipei, Taiwan; Chang Gung University College of Medicine, Taoyuan, Taiwan

^2^ School of Respiratory Therapy, Taipei Medical University, Taipei, Taiwan.

^3^ Department of Pathology, Taichung Veterans General Hospital, Taichung, Taiwan; School of Medicine, Chung Shan Medical University, Taichung, Taiwan

^4^ New Taipei Municipal TuCheng Hospital (built and operated by Chang Gung Medical Foundation), New Taipei, Taiwan

^5^ Airway Disease Section, National Heart and Lung Institute, Imperial College London and Biomedical Research Unit, Royal Brompton Hospital, London, UK.

^6^ Department of Thoracic Medicine, Taipei Medical University Hospital; School of Medicine, Taipei Medical University, Taipei, Taiwan.

Equal Contribution: ^†^ Chun-Hua Wang and Chun-Yu Lo have contributed equally to this work and share first authorship

***Correspondence:** Chun-Hua Wang wchunhua@ms7.hinet.net; Han-Pin Kuo  hpk8828@gmail.com

**Supplemental Figure**


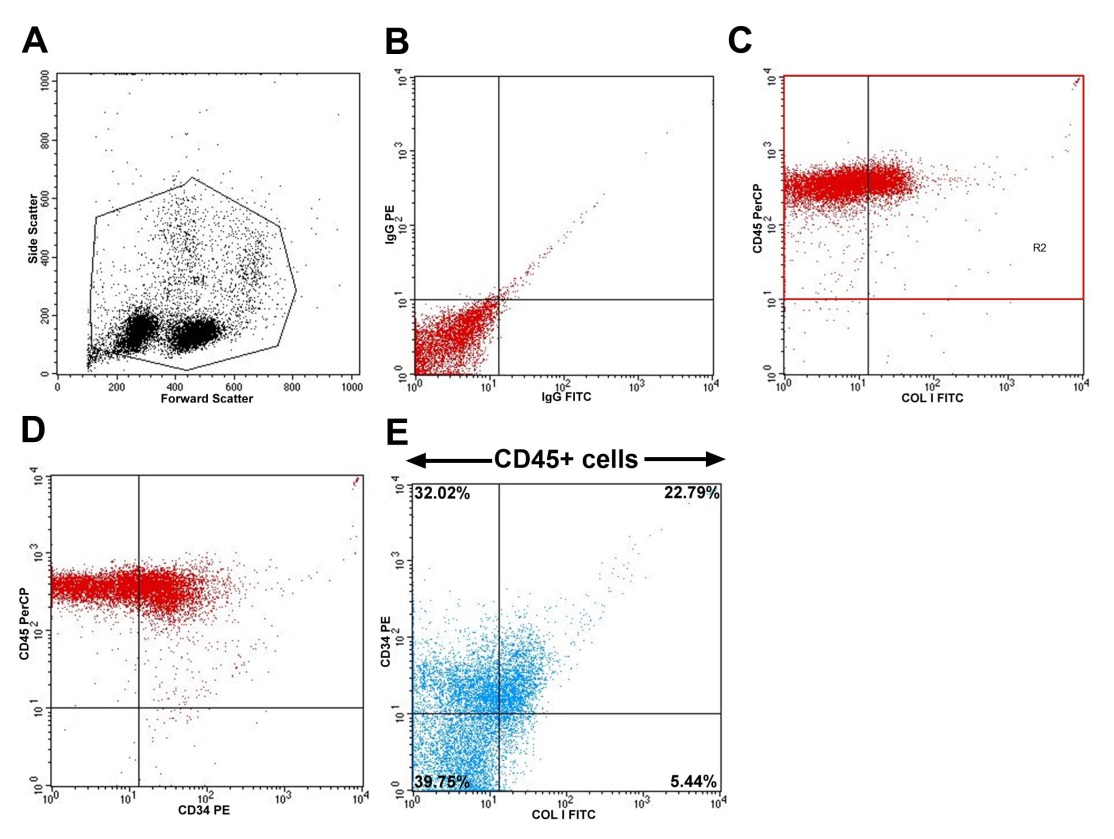


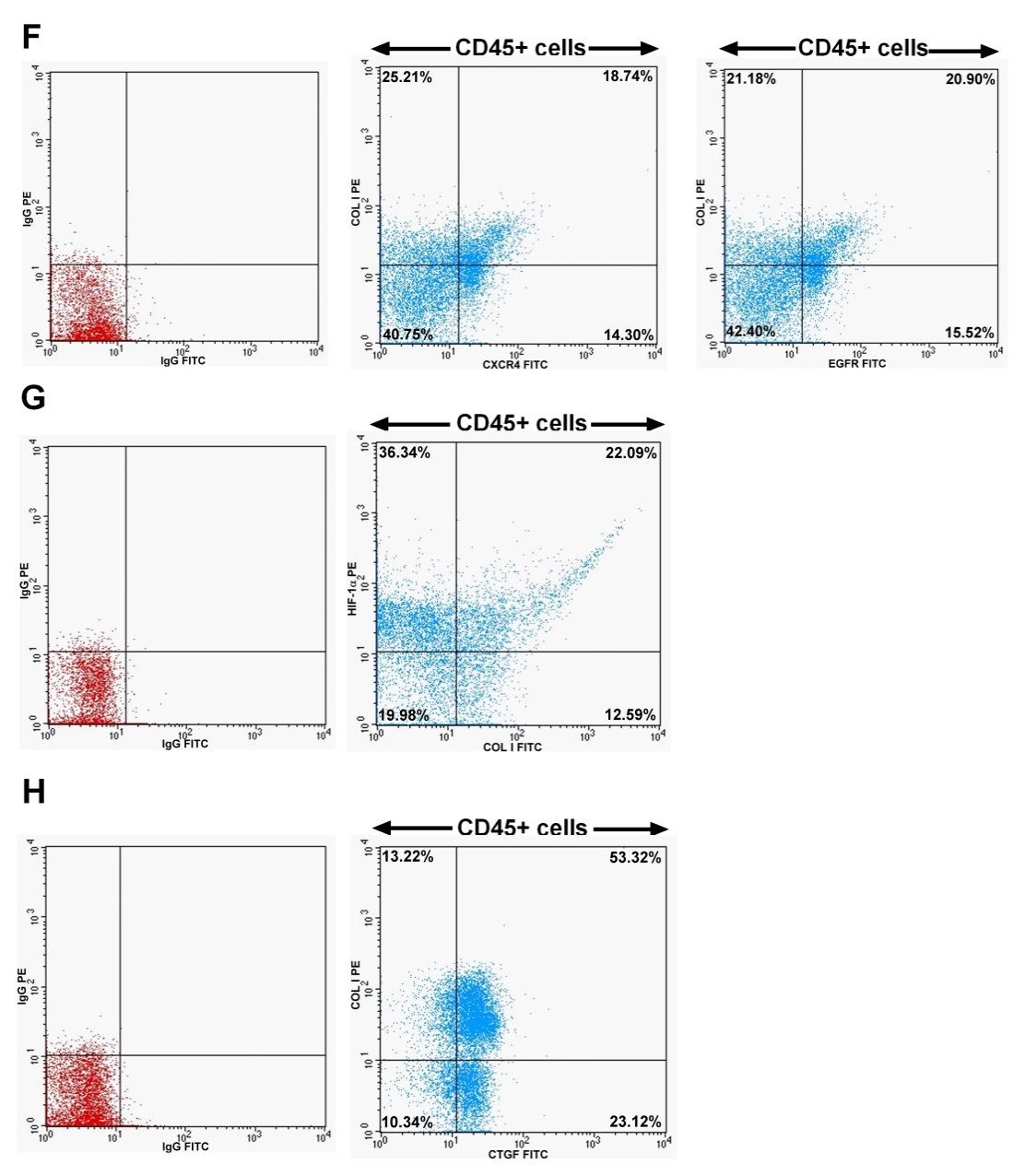


(A) Flow cytometric analysis of non-adherent non-T (NANT) was first gated by region 1 (R1) for analysis of cell population (*horizontal and vertical scales were forward scatter and side scatter*). (B) To identify the fibrocytes, NANT cells were stained with CD34, CD45 and collagen 1 (COL I). (B) Horizontal and vertical lines mark fluorescence intensity greater than background observed with irrelevant phycoerythrin (PE)-, fluorescein isothiocyanate (FITC)-, and peridinin chlorophyll protein (PerCP)-conjugated anti-CD34, anti-COL I, and anti-CD45, respectively, in isotype-matched control antibodies. In NANT cells, some cells coexpressed CD45 and COL I (C) or CD45 and CD34 (D). Cells in the gate of region 2 (R2) were positive expression of CD45 (C). (E) The cells were located in the combined R1 and R2 regions. Circulating fibrocytes showed CD45+ cells that also expressed CD34 and COL I (E). (F) To evaluate the expression of CXCR4 or EGFR in the fibrocytes, the gating process was the same as in the previous description. The horizontal and vertical lines mark fluorescence intensity that was greater than background fluorescence intensity in isotype-matched control antibodies (*left*). Fibrocytes were defined as COL I^+^/CD45^+^ NANT cells by flow cytometry, as previously described (1, 2). The expression of CXCR4 (*middle*) or EGFR (*right*) in the fibrocytes, that coexpressed CD45+ and COL I+ cells, was determined. To identify the expression of HIF-1α (G) and CTGF (H) in fibrocytes, the gating process was the same as for the identification of circulating fibrocytes. Initially, the horizontal and vertical lines that mark fluorescence intensity greater than background fluorescence intensity was set according to isotype-matched control antibodies. The percentage of HIF-1α+ (G) or CTGF+(H) fibrocytes in NANT cells was identified by the coexpression of HIF-1α+ in CD45+ and COL I+ cells.

**References**

1. C. H. Wang, T. H. Punde, C. D. Huang, P. C. Chou, T. T. Huang, W. H. Wu, C. H. Liu, K. F. Chung and H. P. Kuo: Fibrocyte trafficking in patients with chronic obstructive asthma and during an acute asthma exacerbation. *J Allergy Clin Immunol*, 135(5), 1154-62 e1-5 (2015) doi:10.1016/j.jaci.2014.09.011

2. C. M. Weng, B. C. Chen, C. H. Wang, P. H. Feng, M. J. Lee, C. D. Huang, H. P. Kuo and C. H. Lin: The endothelin A receptor mediates fibrocyte differentiation in chronic obstructive asthma. The involvement of connective tissue growth factor. *Am J Respir Crit Care Med*, 188(3), 298-308 (2013) doi:10.1164/rccm.201301-0132OC
